# District nursing and family/whanau assessment practices: A New Zealand study

**DOI:** 10.1002/nop2.1167

**Published:** 2022-01-26

**Authors:** Anna Richardson, Sandra Richardson, Alex McAllum

**Affiliations:** ^1^ Ara Institute of Canterbury Manawa Christchurch New Zealand; ^2^ School of Health Sciences College of Education, Health and Human Development University of Canterbury Christchurch New Zealand

**Keywords:** 15‐Minute Interview, district nursing, family nursing, health assessment, New Zealand

## Abstract

**Aim:**

District Nurses apply specialized nursing knowledge and assessment skills to provide care in New Zealand communities. This study aimed to identify whether District Nurse's (both Registered and supervised Enrolled Nurse's) had knowledge of, and used the 15‐Minute Interview tool, including Ecomaps/Genograms, and if not, what they saw as enablers or barriers to doing so.

**Design:**

Participatory action research was used, following the phases of look, think and act.

**Methods:**

Two pre‐intervention focus groups occurred, two education sessions which introduced the 15‐Minute Interview and four postintervention interviews which explored the use of the tools and their potential use in the future.

**Results:**

District Nurses demonstrated working with families, and the selection of when and where to apply the 15‐Minute Interview.

## 1 INTRODUCTION

District Nursing involves the provision of health and wellness services to individuals and families in their home environments, recognizing that globally populations are ageing with an increased focus on enabling home and community‐based care. The New Zealand Ministry of Health (Ministry of Health [MoH], 2011, p. xii) identifies those most likely to require district nursing (DN) services as aged 75 years or older. In New Zealand, DN services are challenged to increase provision of home rather than hospital care, introduce new technologies, therapies and increasingly complex treatments. DNs are well‐placed to manage these challenges, introducing innovative models of care and service delivery.

Core to DN is the engagement of the nurse with the individual and the family unit. This requires the capacity to gather and share information, and effective documentation and communication within the DN team. Integral to DN documentation and care planning is an effective assessment process. One such process is the Calgary Family Assessment Model (CFAM), developed by Wright and Leahey (2013) reflecting the “ever changing and evolving relationship” between families and the nurses they work with (p. xiv). This model encourages a relational approach to family nursing, enabling nurses to connect across differences, highlighting issues of meaning, experience, race, history, culture and health, with socio‐political systems often emphasized (Doane & Varcoe, 2015; Robinson, 2019). This model enables nurses to attend to the lived experience of the family in their context, recognizing that relationships between patients, families and healthcare providers are central to patient and family care (Bell, 2013).

The CFAM provides a framework that captures the psychosocial context; creating linkages between the cause of the health problem, family members’ beliefs, family dynamics and relationships with health professionals (Duhamel et al., 2015). In an increasingly pressured work environment, nurses are often time poor. Wright and Leahey stated that “family nursing can be effectively, skilfully and meaningfully practiced in just 15 min or less” (Shajani & Snell, 2019, p. 255).

The DN study presented here replicates earlier research undertaken with Public Health Nurses; both centred on introduction of the 15‐Minute Interview, part of the CFAM (Yarwood et al., 2016). The current study population was drawn from nurses working for a DN agency. Potential participants were 36 registered nurses (RNs) undertaking general DN duties, and 13 enrolled nurses (EN, a second‐level registration, under the supervision of RNs). Within this article, when the term DN is used in relation to study participants, this refers to both the registered and supervised enrolled nurses providing health care in the community.

## 2 BACKGROUND

DNs utilize a restorative model of care, which aims to maximize self‐care ability, improving quality of life and self‐esteem with the intent of maintaining optimal functional ability (Senior et al., 2014; Walker et al., 2013). They typically incorporate a socio‐ecological approach to nursing care, based on a holistic, social, and environmental perspective of health, recognizing that social determinants of health may have more impact than medical care on overall health outcomes (McMurray & Clendon, 2015). As part of this approach, DNs acknowledge family members as an integral part of the healthcare team, emphasizing therapeutic conversations as part of this approach (Beierwaltes et al., 2020).

### 2.1 Family and family nursing

The concept of family nursing has developed over the past 20 years, yet research suggests there are still issues in nursing care provision (Bell, 2018; Østergaard et al., 2020). This is due to a variety of factors, including the individual nurse's capacity and knowledge, and structural arrangements in the health system (Eggenberger & Regan, 2010). The definition of family is commonly acknowledged as being “who they [the patient] say they are” (Shajani & Snell, 2019, p. 55). Effective nursing care requires inclusion of the wider family, not only the individual receiving care (Arabiat et al., 2018). Use of a clear conceptual framework encourages the synthesis of data so that family strengths and problems can be identified, and an appropriate nursing plan devised (Shajani & Snell, 2019, p. xiii).

### 2.2 The 15‐Minute Interview

Wright and Leahey, authors of the CFAM, state the experience of health and illness is a family affair (2013). The 15‐Minute Interview was developed as a practical family‐centred approach to determine appropriate health care in a time constrained environment. The five components of the 15‐Minute Interview are outlined in Table 1.

**TABLE 1:** Five components of the 15‐Minute Interview

Manners	Core social skills necessary for interacting with people, whether well or unwell
Therapeutic Conversations	Conversations with families that have purpose
Therapeutic Questions	Questions that enable family health needs to be met
Commendations	Acknowledging a family's strengths and resources
Ecomap and Genogram	The use of the family ecomap and genogram to give a visual illustration of a family's social relationships and networks

The 15‐Minute Interview provides a flexible guide for the nurse, embedded in family nursing and relational practice principles, to conduct a family health assessment. The first element is the therapeutic conversation, where all conversations between the nurse, patient and family are identified as having the potential for healing. They enable the inclusion of family understanding of the experience of illness alongside the nurse's expertize in identifying problems. Therapeutic conversations enable co‐designing of health‐promoting solutions, to jointly manage health issues and alleviate illness suffering (Doane & Varcoe, 2015). The second ingredient is manners; those simple acts of courtesy, politeness, respect and kindness are demonstrated in a society (Shajani & Snell, p. 264). A core demonstration of manners is the nurse introducing themselves to patients and families.

The third ingredient includes the tools of the Ecomap, a diagram of the family's social networks, and the Genogram, a format to capture the history of family behaviour patterns and illustrate the family tree over three generations (Crisp et al., 2017). The ecomap depicts contact with others outside the immediate family which can include sources of support or conflict (Shajani & Snell, 2019, p. 72). Relationships with education, health care, occupation, environment and other systems are acknowledged. The nurse and family can identify social, cultural and economic resources (or their absence) and can be co‐created by the nurse and family. Genograms can illustrate generations of families and highlight repeated patterns and potential for illness, acting as a trigger for the nurse to “think family” (Wright & Leahey, 2013, p. 77). The genogram is a useful tool to visualize complex family structures and identify changes that occur within families between visits.

Ingredient four includes therapeutic questions, those key questions to family members that enable their involvement in family care (Shajani & Snell, p. 266). Nurses are encouraged to develop three contextual questions they ask all families, such as who is the family spokesperson to pass information on to? The fifth element is the use of commendations, where the nurse can feedback positive acknowledgment of a family's strengths, resources and competencies.

## 3 STUDY DESIGN

### 3.1 Aims

The study aimed to identify whether DN’s (both RN and supervised EN’s) currently had knowledge of and used the 15‐Minute Interview tool, including Ecomaps/Genograms, and if not, what they saw as enablers or barriers to doing so.

### 3.2 Methodology and methods

Participatory action research (PAR) was utilized in this study, with the researchers working together with participants in cycles to explore the issues of interest (Koch & Kralik, 2006). Collaborative research with the DNs, acknowledging their clinical expertise and knowledge, enabled the researchers to deepen the shared understanding of DNs work with families. Stringer (1999) outlines three phases of PAR; Look, Think and Act, which was used to structure the research process, and designed to take place in three phases.

Phase one corresponded to the PAR stage of “Look: Building a picture and gathering information”—this involved the establishment of pre‐intervention focus groups, exploring existing DN knowledge of family nursing models and any existing understanding of the 15‐Minute Interview. Phase two corresponded to “Think: Interpreting and explaining”—this allowed for feedback to the DN group of the initial findings generated from the focus groups. Two education sessions were offered, each lasting 30 min, and covering the same core material. This included the formal introduction of the CFAM and the 15‐Minute Interview. The first session was part of an agency education day, with 40 in attendance. The second session was run as a follow‐up opportunity for anyone unable to be present at the first session, with eight participants.

Both of the education sessions began with feedback on the preliminary themes emerging from the focus groups, for confirmation from participants that these were relevant to practice. The CFAM was then introduced, and the 15‐Minute Interview was explained, with an opportunity to practice drawing the ecomap and genogram with a colleague. At this point, the DNs were invited to introduce the model into their practice for a trial period (the intervention). The third phase of the study corresponds to the PAR stage of “Act: Resolving issues”—following the trial period, the DNs were invited to take part in postintervention individual interviews to explore experiences of using the tools and discuss their potential use in the future.

**FIGURE 1:**
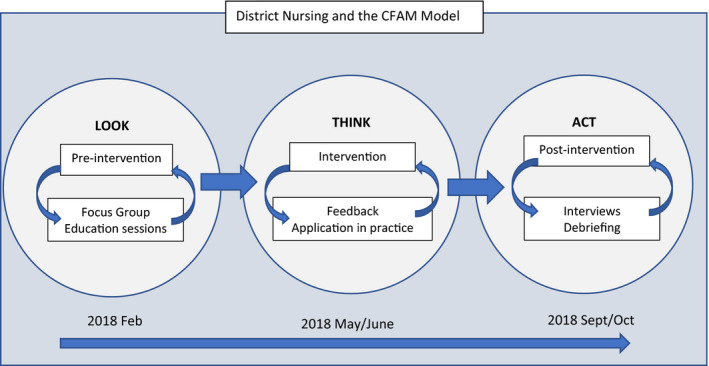
District nursing and the CFAM model

This process is illustrated in Figure 1.

### 3.3 Participants

A convenience sample of nurses were recruited from a single DN agency, from a potential 36 RNs and 13 enrolled nurses. Eight participants took part in phase one (pre‐intervention): three ENs and five RNs whose ages ranged from 25 to over 55 years, with DN experience ranging from one to more than 15 years. Forty‐eight attended the education sessions in phase two and four RN participants completed phase three (postintervention).

For the purposes of this study, DNs are inclusive of the categories of RN and EN. ENs hold NZ registration and work under the direction and delegation of the RN, with the ability to contribute to health assessments, implementation and evaluation of care for patients and their families, while working in the DN service (Nursing Council of New Zealand, 2012).

### 3.4 Ethical considerations

Ethical approval was obtained from the Ara Research Knowledge Transfer Ethics Committee (no. 1759). Organizational locality approval was provided, and permission to present educational sessions and discussion as part of regularly occurring group meetings was given. Informed consent was gained prior to data collection. Anonymity and participant confidentiality were maintained using pseudonyms in transcribing, and the transcriber was required to complete a confidentiality agreement.

### 3.5 Data collection

Data collection occurred across the look, think and act stages of the PAR process. The “look” component began with data collection from two pre‐intervention focus groups. These began with semi‐structured questions, with moderator involvement to clarify, probe and identify experiences and examples, followed by continued discussion in the groups. Examples of questions used included “what is really important for you to know about the family?” and “what difficulties do you experience when working with families?” In line with the participatory nature of action research, the discussion and issues focussed on were mutually generated within the group.

The second component “think” was evidenced with the feedback of findings to the DN team for discussion and review, by means of two interactive education sessions. Researchers fed back findings from the focus group and took notes of the conversations and general points of discussion raised. Participants were invited to complete a short feedback survey, as part of the education session. This asked about their current awareness of the CFAM and if they had used it before. The final component “act” used semi‐structured, individual, face‐to‐face interviews to gather data regarding the experience of utilizing the CFAM in practice. Questions included whether the participants had integrated the 15‐Minute Interview in their practice, and any barriers or facilitating factors they identified. They were asked if they found the model useful to their clinical practice, and why?

### 3.6 Data analysis

Three researchers were involved in the data collection, education sessions and analysis. All audio recordings from the focus groups and interviews were transcribed verbatim. Handwritten notes were taken during the education sessions, and additional recall /reflection entries were collected post these sessions to maintain a data trail. Transcripts were reviewed both individually and collectively using an inductive approach to analyse qualitative data (Thomas, 2006). This approach acknowledges that although the findings are influenced by the research question and the evaluation objectives, they still emerge directly from analysis of the raw data, not from pre‐existing expectations or imposed models. In line with the method of analysis, the researchers read and re‐read the transcripts, at times also referring to handwritten journal notes taken at the time of the focus groups and interviews. Following this, each researcher went through a process of identifying text segments within the data, labelling these to create categories, then comparing these against each other and identifying opportunities for clarification, collapsing and joining of categories, recognizing overlap and redundancies. By this process, the number of categories was gradually reduced, forming summary categories (sub‐themes) and finally synthesized into overarching, simplified themes. Clarity of category coding was confirmed by checking between coders following initial coding of the raw data. Member checking occurred in the form of feedback of the preliminary themes derived from the focus groups at the education sessions, with recognition and acknowledgment of these provided by participants verbally.

### 3.7 Rigour

Trustworthiness was achieved through five criteria: credibility, dependability, confirmability, transferability and authenticity (Cope, 2014). Credibility was enhanced by the participant verification of transcripts, evaluation and summary of the two education sessions through utilization of the same education resources and note taking and recognition of the emerging themes. Confirmability was demonstrated by the utilization of focus group and interview participation, where quotes from the data are utilized to depict emerging themes. The explanation of research processes, such as data collection, with researcher process logs and analysis has promoted dependability (Plummer, 2017). Transferability is determined by the readers of research and their judgement of the ability to apply the findings to their family nursing practice (Connelly, 2016). Authenticity refers to the ability and extent to which the researcher expresses the feelings and emotions of the participants’ experiences in a faithful manner (Polit & Beck, 2012). In the pre‐intervention education sessions, the researchers took the opportunity to present the preliminary findings from the focus groups to check for authenticity. These initial findings were received with interest and discussion about the possibility of utilizing a 15‐Minute Interview in practice.

## 4 FINDINGS

The data generated from the focus groups and the interviews were analysed and synthesized to generate overarching themes. In line with general inductive theory, the researchers were mindful of the evaluation objectives (to determine any existing knowledge relating to use of the 15‐Minute Interview, and what educational needs might be related to introducing this) and were interested in what would emerge from the raw data. Following the pre‐intervention focus groups, the preliminary findings from an analysis of the participant's contributions were fed back to the group at the two education sessions. The emerging themes included definitions of family, DN roles in families, the context of care, family assessment, assessment tools utilized, navigating family issues, communication with family, privilege of family engagement, enablers, and barriers to working with family and relational practice. These were authenticated within the wider group during the discussions held at this time.

Further analysis of the complete data set, combining the follow‐up interviews with the initial focus group transcripts generated the following set of synthesized themes: “Family is who they say they are”; “Navigating complex families and family issues”; The scope of the DN role: “It's the best job in the world.” Each of these overarching themes was informed by several categories/sub‐themes, with these illustrated in Table 2.

Theme One: “Family is who they say they are”

**TABLE 2:** Synthesised themes

Quote (examples)	Category (Sub‐theme)	Synthesised theme
“Family is who they say their family is. Families come in all different forms. It could be your neighbour or a friend. It doesn't necessarily have to be a relation, does it?” (FG P.1) “It's who the actual client refers to as family really, isn't it? As you say, it might not necessarily be blood‐related” (FG P.3) “I think each family's quite individual” (FG P 1)	“Each family's quite individual”	“Family is who they say they are”
“Some clients there's always someone there with them; others you might never see anyone else” (FG P 2) “The only people involved socially … or have interaction with this client … are health services or social services” (Int 4 K) “Or they can be family living in the house ‐ they don't get on, they don't talk.” (FG P 4)	“They don't get on, they don't talk.”	
“Families are really important, because particularly with the difficult clients or the clients that have cognitive decline and they are continuing to live in their own homes by themselves, we need the supports of the family to know exactly what is going [on] behind the scenes” (Int 2 R) “if one member of the whānau's [family] sick, everyone is affected. So that unwellness cannot in my view be limited to the one person, because this particular person who's the client, his behaviours and attitudes – non‐adherence to some of the regimes, medication and dressings really disrupted the whole family unit.” (Int 4 K)	“Families are really important”	“Navigating complex families and family issues”
“…when it's the end of the day and I’ve run out of time and I know I’m already an hour and a half late or whatever. Then when I come into the home, the complications for a couple of my clients are just endless,” “‐ you didn't see the daughter, but the husband and wife would complain of abuse from the daughter, but they didn't feel that they could actually…” “…when you go to somebody and they say, ‘I just want to die, ‐ that's my goal.’”	“Some scenarios can be a little bit scarring”	
“…to honour and acknowledge the person, their efforts, what they're doing, what their family's doing. Because it's their resources, it's their emotional health, it's the drain on them as a family.” “…it's a trust thing as well, because we are strangers walking into someone's house, they are letting us into their house, and they've got to take us on trust really”	“I really want it to be a true partnership”	“The scope of the DN role: ‘It's the best job in the world’”
“We can't judge people. We have to be very non‐judgemental” “Some places you go into, horrible states of things”	“We can't judge people”	
“Sometimes it's just visiting again, and again, and just building that trust. Then once they feel comfortable with you, they'll share”.) “Once they're comfortable, you walk in the door and all of a sudden everything just comes out…”	“I’m present and it's with purpose”	
“…it's so different having a patient in their home environment, in their environment, it's just a whole different ‐ than being in the hospital” “…when you see them at home, you see so much more…”	“it's just a whole different”	
“…because sometimes you feel like you've promised, but you're never sure whether you can deliver” “Then if you get told, ‘well sorry, we can't, that's too much’, you have to go back and say, ‘well look I can't.’” “I feel really pushed for time with each house that I go into. Some days I have up to 20 visits or 20 appointments…”	“it's not always easy.”	

Theme One included two categories: “Each family's quite individual” and “They don't get on, they don't talk.” These illustrated the breadth of family representation identified, with the DNs accepting of (and expected) very fluid outlines of family construction, inclusions and relationships. Illustrations of “Each family's quite individual” identified that definitions of family were not limited to close blood relations or traditional definitions.

“But he had a niece, but she wasn’t the niece…I found out. She was his best friend’s daughter.”

“Family are who the client says they are as well. You’ve got to be quite mindful of that, and it may not be blood‐related, but if they say that they’re family then you have to respect that too.”

In some circumstances there can be an absence or distancing of family, which was recognized in the category “They don't get on, they don't talk.”

“Some clients, there’s always someone there with them; others you might never see anyone else.”

“…for a lot of people, we’re the only person they might see for a week or two on end.”

Theme Two: *Navigating complex families and family issues*

The second overall theme also included two categories—“Families are really important” and “Some scenarios can be a little bit scarring.” This theme encompassed the concepts of working with families and involving them in assessments, while also recognizing the challenges associated with complex family situations. District Nursing was explained as a family affair, where DNs had the opportunity to get to know families well over time. Navigating family issues highlighted a diverse range of concerns, including loneliness, family “interfering,” abuse, fears of patients being “put in a home” and the continuity of care all intertwined in the patient and family stories. An overall sense of complexity in the situations these nurses dealt with was expressed, and the need to interpret, to analyse, to make sense of the myriad chaotic pieces of information and to draw out an understanding and plan.

Under the category “Families are really important,” the DNs expressed their relationship with families, and how they say the opportunities to work together through assessment, information gathering or communication activities.

“…you ring the patient … and they say, ‘oh I have no idea what you’re talking about ‐ you’ll have to talk to my daughter, because she knows.’”

Families were identified as sources of information, and also as needing to receive information, to be kept aware of changes, particularly if not living close by.

“I have emailed people’s daughters or sons overseas, if they’re not here, and they’ve specifically asked us to keep them in touch with what’s going on.”

The second category acknowledges that in dealing with complex situations, these can be uncomfortable for the DN and at times feel as if physically traumatizing, “scarring” because of their intensity. Examples ranged from recognizing clients living in circumstances of poverty, social disarray, isolation and loneliness to finding clients who had died. The ability to interpret behavioural patterns as an expression of loneliness was explained by one DN, who described a situation where a patient deliberately delayed her healing to maintain contact with caregivers and family:

“… Aunty, who was scratching her leg with the knitting needle, I’m sure she only did that because every time anybody said it’s nearly healed, she’d start scratching it again…”.

Other distressing situations included recognition of limited resources, inability to provide all the services or time that the nurse felt was needed or concern when a client disclosed issues of possible abuse. Others noted that individuals, at point of assessment, would indicate that their only “goal” now was “to die.”

“‐ you didn’t see the daughter, but the husband and wife would complain of abuse from the daughter, but they didn’t feel that they could actually…”

Theme Three: “The scope of the DN role: ‘It’s the best job in the world’”

This theme had five categories associated with it, “I really want it to be a true partnership”; “We can't judge people”; “I’m present and it's with purpose”; “It's just a whole different” and “It's not always easy.”

The category “I really want it to be a true partnership” included the DN perceptions of their interactions and relationships with clients, and the roles they identified within the DN position. These included case manager, coordinator, enabler, “empowerer,” facilitator of restorative care and health promoter.

“I really want it to be a true partnership where they’re gonna trust me enough to convey their concerns…”

One DN summarized the essence of the DN role, identifying the significance and depth of the relationship that can develop:

“You’ve had this relationship for three years, and all of a sudden they’re semi‐conscious and …no recognition apart from family members…and it’s that total feeling this person in front of me absolutely trusts me, and that is enormously special.”

One DN described making assessment through family, suspending judgement:

You know they are hiding something. Sometimes it’s just visiting again, and again,

and just building that trust. Then once they feel comfortable with you, they’ll share.

once they’re comfortable, you walk in the door and all of a sudden everything just

comes out, and they tell you exactly what you know has been going on.

Under the category “We can't judge people,” the DNs acknowledged the power they hold, and the responsibility that goes with this; also the challenging situations and clients they had to deal with. They recognized that they may not identify with or approve of people's lifestyle choices, but saw the need to support them to improve their health outcomes.

“…some clients live in a hoarder status but who are we to judge them that that’s not the right way to live? You have to be accepting…”

The third category, “I’m present and it's with purpose,” relates to the DNs communication skills and therapeutic use of self. The nurses were able to describe many examples of establishing effective relationships, not only with clients and families but also with colleagues.

“I can honestly say I’m present and it’s with purpose and I really want to hear what the person is saying. My key thing when I go into the home is that introduction of self and role identifying, ‘cause I haven’t met them before. I want to find out what their concern is and how they want me to work with them’”

The fourth category, “It's just a whole different” refers to the concept of working in the home environment, and the difference this makes in terms of assessment, care provision and relationship building. The nurses described this as a privilege, which was linked to trust, and offered greater opportunities.

“Yet it’s so different having a patient in their home environment, in their environment, it’s just a whole different ‐ than being in the hospital”

“Because you’re looking after people in their own home ‐ it’s such a privilege, and an honour”

The realities of DN were also acknowledged, in the final category: “It's not always easy.” This included recognition of time and resource constraints, exhaustion, moral distress, and process issues.

“Because we’re actually seeing it when we visit them in the home where management, they’re seeing it [from] a statistical point of view.”

### 4.1 The 15 Minute‐Interview feedback

In addition to the analysis of the overall themes, participants who had trialled the Minute interview were specifically asked to feedback on their experience around this. Four DNs shared their perceptions around this.

### 4.2 Determining relevance

Three of the four DNs found that overall, the structure and at least some elements within the 15‐Minute Interview were useful in their practice. Comments related to the usefulness, potential benefit, or lack of applicability and what circumstances affected this. The holistic focus of the 15‐Minute Interview assisted the development of rapport and it was noted that it “brings up other questions.” Responses regarding its capacity to assist with time management were mixed, with some suggesting that in principle it sounded useful (but that they hadn't managed to try it in full), while all still noted that time constraints were an ongoing issue for them. One DN chose not to trial the system, identifying that it offered no additional benefits to the current agency forms.

The importance of including and working with families was highlighted by participants. DNs perceived themselves as being time poor, with pressing demands of patient healthcare needs, while facing complex health and family situations. Despite the challenging workloads, DNs continued to value the role they played, and the opportunity to interact with and support family/whānau. The prompt with the CFAM process to provide commendations to family were considered helpful in promoting patients/family to keep control of their care. For DNs who work with Māori and value a whānau (family) approach, the model is seen to align well.

### 4.3 Using the ecomap and genogram

The genogram was identified as being used most often. For one participant, this was in conjunction with an existing process, the InterRAI assessment. Both processes are ways of showing links and relationships between individuals, of illustrating family connections. The 15‐Minute Interview was also seen to align to the Hui Process, a Māori framework to guide clinical interaction and engagement of Māori patients and whanau in the health services (Lacey et al., 2011, p. 72).

## 5 DISCUSSION

Overall, the findings from our research align with much that is identified in wider family research. The theme of “Family is who they say they are” identified broad and inclusive representations of what constitutes family. The definitions offered align with concepts of self‐identification and interdependence which underpin many family theories, and the recognition that “family” as a construct may be built on connections through marriage, blood ties, adoption, friendship or other (Doane & Varcoe, 2015; Wright & Leahey, 2013). Such theoretical understandings and broad definitions are useful to DNs, supporting their ability to collaborate with the family, regardful of each family form.

The theme of “Navigating complex families and family issues” recognizes the relationships that DNs develop with family and the importance placed on understanding and involving family in decision‐making and support for the patient. DNs valued their engagement with families and spoke of the “privilege of home visits” while acknowledging the challenges associated with navigating complex families and addressing family issues. Our research highlighted that a person's illness is often a family illness and that the developmental stages of each family can be complex. Family can affect the illness journey, often seen when a family member's health is failing in older adulthood, with family issues coming to the fore. The DNs described the use of therapeutic conversations and commendations when working with families, even when not recognizing these as specific elements from the CFAM. The participants may not have identified that their actions matched the theoretical model, yet they focussed on family strengths, supporting physical, emotional and spiritual wellbeing. The DN’s spoke of taking the “long game” acknowledging that they work alongside the patient and family to provide care, over time.

The theme: “The scope of the DN role: ‘It's the best job in the world’” allowed the DN’s to talk about the use of tools, and the various roles they needed to undertake, seeing the technical as well as the practical elements of their position. They described taking on case manager, coordinator, enabler, and “empowerment” roles. This reflects international literature, identifying the increasing expectations being placed on DNs, and the associated stresses and impacts (Duncan, 2019). Given the time scarcity that participants also identified, the question remains whether there is sufficient resource to enable all of these roles to be undertaken effectively. DNs are typically described as providing preventative care, with the aim of achieving avoidable admissions to hospital and expediting early discharge. The support of DNs enables patients with short term and long‐term conditions, to concentrate on their recovery and health care in their own homes. However, participants in this study identified the conflict arising from multiple roles and time constraints, difficulties being able to spend as much time as they might have wanted to with individuals and their families, and the increasingly complex nature of the workload they dealt with. While the introduction of a tool such as the 15‐Minute Interview may add to the ability to provide holistic care, and early identification of circumstances amenable to intervention, the expectation to see high volumes of patients in a day limited their capacity to make use of this.

DNs are being constantly challenged to take on new activities, higher workloads, and to incorporate new technologies into their practice. Within this study, the DNs were asked to trial the 15‐Minute Interview as part of their assessment practice. Despite indicating a general willingness to do so, very few ultimately followed through and reported on the experience, most commonly citing time constraints. This has implications when considering the capacity for the workforce to continue to introduce or change aspects of practice. When analysing the feedback of those who did trial the model, it was apparent that while there was potential for the family assessment tool to be incorporated alongside other processes routinely used by the DNs, this was seen as requiring time and resource that was not always available. Even where processes (such as the CFAM model) were identified as useful in principle, the opportunity to incorporate them into practice was not always present. This resulted in the DNs selectively choosing elements, typically parts of the ecomap or genogram, which they then adapted for use. This demonstrates the prioritization and adaptation skills used by DNs, but also the limitations and risks associated with informal and partial adoption of systems. DNs described long lists of patients to care for each day and some preferred to use the brief agency‐based record of contact with the patient, especially for brief DN/patient encounters. There was acknowledgement that repeated use of the model might make it easier to use. Leahey and Wright (2016) support the grounding of nurses in family conceptual practice models as the application of them assists the nurse to develop the skills and competencies to help families more efficiently and easily.

The usefulness of the 15‐Minute Interview was summarized as assisting the DN to collaborate with the patient and family and explore the support systems the patient might have. As many of the patients were older adults, the 15‐Minute Interview assisted identification of potential social isolation and provided an opportunity to understand the social dynamics in the family. Repeating the application of the framework over time assisted identification of health issues, such as memory loss. Participants found that the 15‐Minute Interview was “more personal” than the structured InterRAI framework and family members appeared “totally comfortable” being interviewed utilizing the tool. The genogram was valued for the ability to highlight hereditary components of health and illness. Overall, while it took extra time to use the 15‐Minute Interview, the participants found it was “putting in time, to save time,” meaning more effective and efficient interviews with the patient and family subsequently. Nurses are increasingly urged to engage with families—to “think family” (Duhamel et al., 2015; Eggenberger et al., 2011). This is particularly important for indigenous peoples, and DNs working with Māori whānau (family) in New Zealand are expected to include whānau in the patient's care. Māori perceive themselves as a collective, with the inclusion of whānau considered essential to health assessment and culturally appropriate nursing care (Pitama et al., 2014). Taking a whānau approach enables the comprehensive assessment of a Māori patient in the community. The participants recognized that the 15‐Minute Interview was compatible with the holistic Māori framework of Te Whare Tapa Wha (translated as the four cornerstones of the house, including spiritual, mental, physical, and extended family dimensions (Durie, 1994), and the Hui process. The Hui process was used with Māori patients and whānau and is a structured model that ensures clear introductions of the nurse, patient and whānau and emphasizes the importance of making a connection for working together in the future (Lacey et al., 2011). This echoes Wright and Leahey's emphasis on therapeutic conversations and making commendations, with the addition of the nurse sharing a little of themselves, in a professional manner, to make key connections with the patient and whānau. The connection could be sharing of the nurse, patient and whānau place of upbringing or residence.

When considering future practice of the DN, it is likely that additional DN responsibilities will be added, or existing scopes expanded rather than reduced, alongside the ageing population and push towards community care. West (2014) discusses the palliative care roles that DNs hold in New Zealand and suggests there will be a growing demand for care of people dying in their homes, as part of a world‐wide trend, alongside increasing care of patients with dementia (Coldrick & Crimmons, 2019). Stajduhar (et al., 2010 as cited in West, 2014) suggest that DNs have a delicate balance of the provision of care to ensure the health needs of the patient and family are met. As Leahey and Wright (2016) have identified, illness is a “family affair” and the use of the 15‐Minute Interview assists the DN to apply theory to practice. The purpose of the 15‐Minute Interview is a rapid interview, which can assist the DN to develop an assessment efficiently and make a difference to the patient and family illness experience and indeed, the DNs nursing satisfaction.

### 5.1 Strengths and limitations

The strength of this study is the contribution to the body of knowledge in terms of a “snapshot” of DN in New Zealand and the use of family nursing models. There is a dearth of current international literature related to the use of family models in district or community nursing or the application of Wright and Leahey's CFAM. However, the limitation is the small number of participants. The patient load was identified by participants as the key contributing factor for declining to participate in the research or to utilize the CFAM. However, the data from the study can be helpful in the context of family nursing and how nurses can involve family in district nursing. Further research in family nursing in the district and other community nursing practice is needed.

## 6 CONCLUSION

Our research has provided useful insights into DN family practice in New Zealand. DNs described a broad and inclusive definition of family structures, valuing family inclusion and engagement. Collaboration, support, negotiation and shared decision‐making were described by DNs working to engage each family.

The CFAM, particularly the 15‐Minute Interview, were seen as potentially useful tools relevant to several areas of practice; however, time constraints and pressures of practice were identified as barriers to utilizing these approaches in practice. The DNs described utilizing several of the components of the 15‐Minute Interview, sometimes without realization, such as therapeutic questioning and commendations. Utilizing the complete CFAM was acknowledged as enabling analysis of the patient and family structural, developmental and functional capabilities, highlighting patient and family coping in chronic illness, stress and level of family supports. The concept of family and the role of DNs as family nurses was evident in this study. Skilled practice demonstrated that even where the individuals may not have recognized their practice as being “family nursing” their actions matched the theoretical constructs.

This study highlighted the constraints to introducing new tools and approaches into already pressured workloads, even when these have potential to improve efficiency or engagement. The time needed to gain familiarity, and to apply new interventions needs to be balanced against future benefits; this has significance in terms of the range of additional tasks being asked of DNs. DNs described the challenge of balancing the provision of care to ensure the needs of the patient were met, with the time given and the patient allocation for the day. If the CFAM were to be introduced into district nursing, personal, organizational and professional barriers would need to be addressed.

## CONFLICT OF INTEREST

The authors declare no conflict of interest.

## Data Availability

The data that support the findings of this study are available from the corresponding author upon reasonable request.
